# Validation of Pose Estimation Models for Automated Kickboxing Technique Analysis Using 2TRAX3

**DOI:** 10.3390/s26195997

**Published:** 2026-09-22

**Authors:** Samir Duvelek, Dominik Peter Hoelbling, Roland Breiteneder, Benedikt Mitter, René Baranyi, Thomas Grechenig

**Affiliations:** 1 Research Group for Industrial Software (INSO), Vienna University of Technology, 1040 Vienna, Austria; samirduv@gmail.com (S.D.);; 2 Software and Digital Systems Engineering, University of Applied Sciences Wiener Neustadt (FHWN), Biotech Campus Tulln, Konrad Lorenz Straße 10, 3430 Tulln, Austria; 3 Institute of Software Engineering and Artificial Intelligence, TU Graz, Inffeldgasse 16b/II, 8010 Graz, Austria; 4 RISE Institute of Technology (RIT), SSS District, Ongole 515134, India

**Keywords:** pose estimation, biomechanics, martial arts, double side kick, motion capture, Bland–Altman analysis, temporal phase analysis, computer vision

## Abstract

Biomechanical feedback can improve sports technique, but the 3D motion capturing gold standards typically used to generate such feedback are rarely available in everyday training. This study evaluates how the choice of pose estimation model affects automated movement analysis of the double side kick in Pointfighting, Kickboxing using ordinary 2D side-view video. In doing so, the study aims to determine whether advances in pose-estimation models translate into meaningful improvements in complete, sport-specific movement-analysis systems under realistic training conditions. The analysis compared MoveNet, YOLO11x-Pose, ViTPose-L, and RTMPose-L on 27 videos recorded under varying lighting conditions. Seventeen movement characteristics extracted from the videos were compared with 3D motion-capture measurements of the same kicks. Friedman tests with Wilcoxon signed-rank post-hoc tests showed that model choice significantly affected phase-duration errors (p<0.01, Kendall’s W=0.150) and femur-angle errors (p<0.01, Kendall’s W=0.174), but not leg-spreading angles or distance- and height-based characteristics. ViTPose-L achieved the lowest median errors for phase durations (0.043 s, IQR 0.035 to 0.062 s), femur angles (6.95°, IQR 4.35 to 11.65°), and leg-spreading angles (6.22°, IQR 5.08 to 10.04°), while RTMPose-L had the lowest median error for distance- and height-based characteristics (6.09 percentage points, IQR 3.84 to 9.10). Bland–Altman analysis showed generally small systematic biases, but acceptable agreement with the 3D reference system was achieved only for a small subset of the evaluated characteristics. The runtime benchmark, performed on the tested local hardware, further demonstrated substantial differences in computational efficiency, with mean processing speeds ranging from 6.50 ± 0.10 FPS for ViTPose-L to 66.87 ± 0.36 FPS for MoveNet. These findings indicate that, for 2D technique analysis of rapid sports movements from side-view recordings that meet minimum quality requirements, ViTPose-L and RTMPose-L are preferable when accuracy and keypoint completeness are prioritized, whereas MoveNet is preferable when runtime performance on comparable hardware is the main requirement. Therefore, the choice of pose-estimation model is strongly use-case dependent, and automated 2D analysis should be interpreted as approximate feedback for selected characteristics rather than precise individual-level biomechanical measurement.

## 1. Introduction

Technique analysis helps athletes execute movements correctly and can thereby improve competitive performance [1,2,3]. It supports self-improvement by revealing weaknesses and enabling targeted training, even guiding tactical preparation by revealing exploitable weaknesses in an opponent’s technique [4]. Biomechanical and kinematic research has analyzed sports technique for decades [5].

However, this type of analysis requires costly equipment, substantial time and expertise. Complex techniques consist of many small body movements, and identifying the changes an athlete needs for improvement requires a detailed, multidimensional view of the motion [6,7].

Wearable sensor technologies are increasingly being used for performance analysis in sports [8]. Inertial measurement units (IMUs), in particular, have also been investigated for performance analysis in combat sports [9]. While such systems are portable and suitable for use outside laboratory settings, wearable technologies still commonly require dedicated hardware and are associated with additional costs [8]. In combat sports, high-impact and high-velocity movements can introduce additional challenges related to sensor placement, attachment, and displacement [9,10]. Approaches using readily available hardware also exist. For example, Pezenka and Wirth used the inertial sensors of a common smartphone to measure punch and kick velocity [11]. However, this approach targets a specific performance variable rather than providing a multidimensional analysis of the technique as a whole.

3D motion-capture systems such as the one from Vicon^®^ can extract analysis characteristics semi-automatically and, combined with expert knowledge, provide athletes with specific guidance for their training [7,12]. However, a regular training environment rarely provides access to such systems because they require expensive equipment, controlled recording conditions, technical expertise, and time-consuming processing [6,7,12].

A fully automated analysis based only on 2D video may, therefore, provide an even more accessible solution for everyday training, as it requires only a video recording and no sensors or devices to be attached to the athlete. However, previous pose-estimation approaches have often been evaluated under conditions that do not fully reflect real sporting environments, where factors such as occlusion, background complexity, and recording conditions can affect performance [13]. To enable seamless integration into everyday training, such a system must, therefore, combine high analytical accuracy with sufficient computational performance to provide feedback with minimal delay and operate under the less controlled recording conditions encountered in regular training environments.

### 1.1. State of the Art in 2D Video-Based Movement Analysis

The application of camera-based human pose estimation in sports and physical exercise has received increasing research attention in recent years, with deep-learning-based approaches being investigated across a growing range of sports and movement-analysis tasks [13,14]. Markerless vision-based analysis has also been applied in combat sports, for example to estimate upper-extremity kinematics during boxing [15], although that study used a multi-camera setup rather than the single-camera recording examined here. Several studies have validated movement parameters extracted from 2D video against 3D reference systems, including continuous sagittal-plane running kinematics on a treadmill [16], lower-extremity joint angles during a single-leg squat [17], posture and coordination measures during a floor-to-waist height lifting test [18], and baseball pitching mechanics from a single camera [19]. These tasks differ from the double side kick, the kickboxing technique investigated in the present study and described below, in two respects. First, in each of them the performer remains approximately in place, the movement is largely confined to the recording plane whereas in the double side kick the athlete translates through the frame, the kicking leg rotates out of the sagittal plane, and the technique is executed at high angular velocity. Second, the parameters reported in these studies are either continuous joint-angle waveforms or values taken at a single event, whereas the characteristics of the double side kick are defined relative to a set of predefined movement nodes that must first be identified from the keypoint trajectories according to fixed rules [12]. Node identification is, therefore, an additional error source and it constrains the achievable accuracy independently of the quality of the pose estimation.

Furthermore, the cited task-specific validation studies do not examine whether the choice of pose-estimation model affects the reported agreement, as each validates a single model. Direct comparisons of pose-estimation models do exist, but they evaluate models either at the level of image keypoints [20], against a reference system on generic locomotor and exercise tasks [21], or for a comparatively narrow outcome such as joint range of motion [22]. Less attention has been given to the effect of model choice on the output of a complete, sport-specific technique-analysis pipeline.

Taken together, these studies demonstrate the potential of 2D video-based movement analysis, but less is known about how pose-estimation models perform within complete sport-specific pipelines under the less controlled recording conditions of everyday training [13].

### 1.2. Double Side Kick

The double side kick is a Pointfighting, Kickboxing technique in which the athlete executes two consecutive side kicks with the same leg during one continuous movement sequence. Its execution requires coordinated preparation, knee lift, kicking-leg extension, and retraction. Because the technique is fast and consists of several closely connected movement phases, its analysis depends on the precise identification of key movement nodes and the extraction of temporal, spatial, and angular characteristics.

Previous biomechanical studies defined the double side kick as a sequence of characteristic movement nodes and phases and investigated temporal, spatial, angular, and velocity-based indicators of its execution. These included phase durations, normalized measures of knee height and knee-to-shoulder distance, femur and leg-spreading angles at selected movement nodes, and kicking-leg velocities. The measured values of multiple characteristics were correlated with the athletes’ competitive success [12,23,24]. The identified movement structure and performance indicators provide the biomechanical foundation for the automated analysis developed in the present study.

### 1.3. Evolving Pose Detection

Recent years have produced specialized software and hardware that use various sensors to address problems in health and sport [25,26,27], including systems such as the one presented here. At the same time, artificial intelligence models, including the pose-estimation models underlying this system, have improved substantially [28]. These developments raise a practical question: what do improvements in model accuracy mean for the specialized systems that rely on these models?

This question is especially relevant in real training environments, where facilities are rarely designed for filming and coaches and athletes cannot always control the lighting, camera position, background, or recording quality. These uncontrolled conditions provide a demanding but informative test case because they reveal whether models tested and optimized on standardized benchmark data [29] actually improve real-world applications. However, practical usefulness depends not only on measurement accuracy but also on computational performance, as efficiency is an established component of usability [30].

### 1.4. Aim, Research Questions, and Hypotheses

The aim of this study was to evaluate how the choice of pose-estimation configuration affects the accuracy and practical suitability of automated 2D movement analysis of the double side kick. The study compared configurations based on MoveNet, YOLO11x-Pose, ViTPose-L, and RTMPose-L within the same subsequent movement-analysis pipeline. Movement characteristics extracted from 2D side-view videos were compared with corresponding 3D motion-capture measurements from previous work [12].

The study addressed the following research questions:

1. How well do the movement characteristics extracted from 2D videos agree with the corresponding 3D reference measurements?
2. Does the choice of pose-estimation configuration significantly affect the absolute error of the extracted movement characteristics?
3. Does trajectory anomaly detection improve the agreement between the 2D analysis and the 3D reference system?
4. How do the evaluated pose-estimation configurations differ in runtime performance and keypoint completeness?

Based on these research questions, the following hypotheses were tested:

**H1.**  *The pose-estimation configuration has a significant effect on the absolute error of the extracted movement characteristics when compared with the 3D reference system. This hypothesis was tested separately for the predefined movement-characteristic groups.*

**H2.**  *Enabling trajectory anomaly detection significantly reduces the absolute error of the extracted movement characteristics compared with anomaly detection disabled.*

## 2. Materials and Methods

This section describes the dataset used for the validation, the recording setting, the automated analysis system, and the extracted movement characteristics. It also explains the statistical analyses used to compare the pose-estimation models, evaluate anomaly detection, assess agreement with the 3D reference system, and benchmark runtime performance.

### 2.1. Participants and Dataset

The original dataset comprised 44 double-side-kick recordings from 44 athletes with varying levels of competitive success. The recordings were obtained in a previous biomechanical study [12]. They were produced with the video equipment available at that time and were not intended for markerless computer-vision analysis.

Seventeen recordings could not be included in the present analysis because their image quality, particularly the poor lighting conditions, prevented sufficiently reliable initial pose detection. As a result, complete and reliable keypoint trajectories, which form the basis for all subsequent movement-node detection and characteristic extraction, could not be generated. Consequently, no further movement analysis could be performed for these recordings. The final dataset, therefore, comprised 27 kick executions from 27 athletes.

### 2.2. Experimental Setup of Dataset Study

The videos show the double side kick from a side view at 24 fps under varying lighting conditions. The analysis compared the characteristics extracted from the 2D videos with the 3D motion-capture measurements of the same kick executions from the earlier study and treated those measurements as the gold standard [12]. The 3D motion-capture system recorded at a sampling rate of 250 Hz. The 3D reference characteristics had already been extracted in the previous study, while the same characteristics were independently derived from the corresponding 2D recordings. The comparison was, therefore, performed at the level of the resulting movement characteristics rather than between temporally synchronized 2D and 3D frames.

### 2.3. System Description

The present study builds on 2TRAX3, an automated movement-analysis system developed in previous work to extract predefined characteristics of the double side kick from 2D side-view video [31]. For the present study, the system was substantially extended through the integration of four interchangeable pose-estimation models, a revised node-detection algorithm, and a modified trajectory anomaly-detection module.

For each video, the system requires the athlete’s height and the identification of the kicking leg as input. A pose-estimation model then detects keypoints on the athlete’s body in every frame. The processing pipeline analyzes the resulting keypoint trajectories, applies the established rules to identify the frames corresponding to the predefined technique nodes, and calculates the selected movement characteristics at those nodes. Once the video, athlete height, and kicking leg have been provided, the remaining analysis is performed automatically without manual frame selection or annotation. The system was developed through an iterative prototyping process based on requirements derived from a series of interviews with a kickboxing domain expert.

Adapter interfaces integrated the pose-estimation configurations into the pipeline and enabled configuration substitution without changes to the remaining analysis code. This allowed the evaluated configurations to be compared within the same downstream movement-analysis pipeline.

Although all evaluated configurations were integrated through the same adapter interface and returned the same 17 COCO-format keypoints, they differed in their input framing. MoveNet and YOLO11x-Pose were evaluated as full-frame configurations, whereas RTMPose-L and ViTPose-L were evaluated as top-down configurations using RTMDet-m person detection and athlete cropping before pose estimation. Therefore, the primary comparison should be interpreted as a comparison of complete pose-estimation configurations rather than isolated pose-estimation backbones. To address the potential influence of input framing, additional variants of MoveNet and YOLO11x-Pose were evaluated in a secondary sensitivity analysis using the same RTMDet-m person detector.

### 2.4. Variables

The extended system extracted the 17 movement characteristics defined in Table 1. These characteristics represent the temporal, spatial, and angular indicators selected for the present study; the velocity-based indicators examined in previous biomechanical work were not included. The corresponding movement nodes and phase definitions are presented in Table 2 and are based on previous studies of the double side kick [12,23,24].

For each pose-estimation configuration, the absolute error equaled the absolute difference between the value obtained from the 2D video analysis and the corresponding 3D reference value. Because every configuration processed the same kick executions, the resulting errors constituted paired repeated measurements.

### 2.5. Statistical Analysis

All statistical analyses used a significance level of α=0.05. All inferential tests were two-sided. Because this was a retrospective validation study based on all available recordings that met the inclusion criteria, no formal a priori power analysis was performed. The sample size was, therefore, fixed by data availability and video quality.

To address the effect of pose-estimation configuration on the extracted movement characteristics, the statistical comparison evaluated the configurations separately for each movement-characteristic group: phase durations, femur angles, leg-spreading angles, and distance- and height-based characteristics. The analysis used the median absolute error within each group for every subject and configuration. Following the non-parametric approach for comparing multiple classifiers described by Demšar [32], a Friedman test compared the configurations’ error ranks [33]. Kendall’s *W* was reported as the effect size for each Friedman test.

When the Friedman test indicated a statistically significant configuration effect, pairwise post-hoc comparisons used Wilcoxon signed-rank tests [34]. The Holm correction adjusted the resulting *p*-values for the six pairwise configuration comparisons within each movement-characteristic group [35]. Matched-pairs rank-biserial correlations were reported as effect sizes for the pairwise Wilcoxon signed-rank tests.

Each comparison included only complete paired observations. A subject entered the test for a given characteristic group only when every compared model produced valid values for all variables in that group. Missing values occurred when a characteristic could not be computed from the pose-estimation output at one or more of the identified movement nodes. These observations were excluded because a paired comparison requires a valid result from every model for the same kick execution. All exclusions occurred with MoveNet, while the remaining three models produced valid values for the same executions. One athlete was affected across the phase-duration, femur-angle, and leg-spreading-angle groups, one further athlete in the femur-angle group, and three further athletes in the leg-spreading-angle group. The distance- and height-based characteristics were available for all recordings. Consequently, the sample size varied by characteristic group, as reported in Table 3.

Trajectory anomaly detection was intended to identify and correct implausible keypoint trajectories before movement-node detection and characteristic extraction. Its effect was evaluated as a secondary exploratory analysis. For each pose-estimation configuration and movement-characteristic group, the absolute errors obtained with anomaly detection disabled were compared with those obtained with anomaly detection enabled using paired Wilcoxon signed-rank tests. Subjects whose error was identical under both conditions carried no information for the signed-rank test and were, therefore, excluded from the signed-rank calculation. The effective sample size of each comparison, therefore, equaled the number of non-zero paired differences. Because this number was as low as one for several comparisons, the tests used the exact null distribution rather than the normal approximation. The resulting *p*-values were adjusted using the Holm correction across all 16 anomaly-detection comparisons. Due to the low number of informative pairs in several comparisons, this analysis was interpreted descriptively and not as a confirmatory hypothesis test.

To answer research question 1, Bland–Altman analysis [36] was used to evaluate agreement between the video-based measurements and the 3D reference values for all four pose-estimation configurations. For each dependent variable, paired differences were calculated as the video-based value minus the corresponding 3D reference value. Shapiro–Wilk tests were used to assess the normality of the paired differences. The detailed Shapiro–Wilk results are reported for the two primary agreement configurations, ViTPose-L and RTMPose-L, in Appendix B. For these configurations, departures from normality were observed for several characteristics. Nevertheless, the conventional 95% limits of agreement, calculated as the mean difference ±1.96 standard deviations of the differences, were retained [36]. Departures from normality were reported explicitly and considered when interpreting the results. Given the small sample size, with at most 27 paired observations depending on the characteristic and configuration, 95% confidence intervals were additionally calculated for the mean bias and the limits of agreement to indicate the uncertainty of these estimates. Agreement was evaluated by comparing the limits of agreement with the predefined study-specific acceptable limits for each characteristic.

For the angle characteristics, a study-defined tolerance of ±20° was specified before the agreement analysis, based on error magnitudes of approximately 20° reported in previous monocular movement-analysis studies [21,37]. Agreement was considered within this tolerance only when both 95% limits of agreement lay between −20° and +20°. Because the double side kick is a rapid, multi-phase movement and no established biomechanical threshold for acceptable agreement was available, this tolerance was interpreted as an exploratory, study-specific criterion for approximate feedback rather than as a generally applicable limit for precise biomechanical measurement.

No directly comparable limits were available for the duration, distance-, and height-based characteristics. The analysis, therefore, retained the numerical study-specific agreement limits from the previous work [31]. A common relative range-based criterion was used for these non-angular characteristics, with the limit defined as half of the observed range of the respective 3D reference characteristic in the present sample.

For the phase-duration characteristics, the video frame rate limited how strict this criterion could be. Because movement nodes were identified at individual frames, limits smaller than one frame would not be practically meaningful. At 24 fps, one frame corresponds to approximately 41.7 ms. This is especially relevant for KI1, for which the retained 0.050 s limit is only slightly larger than one frame.

For the distance- and height-based characteristics, no directly comparable biomechanical or task-specific agreement thresholds were available. The same relative range-based criterion was, therefore, applied to these characteristics, with the understanding that the resulting limits are study-specific and data-dependent. Accordingly, these limits should be interpreted as broad exploratory criteria for approximate feedback in the present study, not as established biomechanical thresholds for acceptable agreement. Because they depend on the range of values in the present sample, they may differ in other athlete populations.

### 2.6. Performance Benchmark

To answer the fourth research question, a performance benchmark compared the pose-estimation configurations in terms of runtime performance and keypoint completeness. The benchmark measured the configurations under conditions that closely matched the deployed system: it used the same code paths as the automated analysis and timed the complete per-frame pipeline, including streaming video decoding with FFmpeg [38] and pose estimation for each frame. The reported runtimes exclude model loading.

The benchmark evaluated each configuration in a separate Python 3.11 process and repeated the measurement five times per configuration. To limit thermal throttling, each repetition used a shuffled configuration order, and a 180 s cool-down period separated consecutive benchmark processes. The machine remained connected to power with the lid open and all background applications closed. The benchmark did not force CPU or GPU execution; each adapter automatically selected the available backend.

In each run, the first 15 frames served as a warm-up and did not contribute to the timing. The benchmark then processed all frames of the selected kick videos and recorded two runtime measures: the complete processing pipeline, including video decoding, and, as a second measure, the isolated pose-estimation stage. The complete-processing measure served as the primary measure because it more closely reflects the operating conditions of the automated system.

The benchmark used the same kick videos as the configuration comparison. For each video, it recorded the number of frames, resolution, frame rate, wall-clock runtime, accumulated pose-estimation runtime, end-to-end frames per second, and pose-estimation frames per second. The same inference pass also recorded pose-estimation stability statistics: the number of frames with complete COCO-17 [29] keypoint detections, the number of frames containing all task-relevant keypoints, and the number of frames in which the configuration detected no person. The task-relevant set comprised the left and right ankles, hips, knees, and shoulders, together with the nose, because the analysis requires all of these keypoints.

After all repetitions, the analysis aggregated the results across runs. For each configuration, the summary reports the total number of processed frames, the overall end-to-end frames per second, the mean and standard deviation of end-to-end frames per second across runs, the overall pose-estimation frames per second, and the percentage of frames with incomplete task-relevant keypoints. These values support a practical comparison of configuration runtime and detection stability.

## 3. Results

This section describes the implemented automated-analysis pipeline, compares the pose-estimation configurations, and evaluates the 2D results statistically against the 3D gold standard.

### 3.1. Implemented Analysis Pipeline

The analysis pipeline consists of several processing steps. First, the system processes every frame using the selected pose-estimation configuration, which returns the positions of the 17 COCO-format keypoints [29] for the detected athlete. An adapter interface integrates four pose-estimation configurations and allows the system to switch between them without modifying the remaining analysis code. The study evaluated configurations based on MoveNet [39], YOLO11x-Pose [40], ViTPose-L [41], and RTMPose-L [28]. The system applied a minimum confidence threshold of 0.20 to all configurations and treated any keypoint below this threshold as missing in that frame. The decision to use a relatively low confidence threshold was made because the study aimed to investigate the effect of the pose-estimation configurations on the complete analysis pipeline. A lower threshold was preferred because it retained potentially erroneous detections for subsequent processing, allowing the types of errors produced by the pose-estimation configurations to be examined rather than removing these detections entirely through a higher threshold. This also allowed the robustness of the subsequent anomaly-detection procedure to be evaluated in the presence of low-confidence detections. Linear interpolation replaced missing keypoints in the angle calculations, whereas node-detection rules that depend on keypoint coordinates ignored missing values. The task-relevant keypoints comprised the nose and the athlete’s left and right shoulders, hips, knees, and ankles. The kick analysis used the ankles as anatomical approximations of the heel positions.

The distance- and height-based characteristics are normalized to a neutral standing posture, which the system determines automatically from the frames preceding the initialization node. Among these frames, it selects the one in which the heel, knee, hip, and shoulder keypoints of the standing side are most closely vertically aligned, quantified as the minimum sum of their horizontal offsets. In this frame, the trochanter major height is taken as the vertical distance between the heel and hip keypoints, and the knee-to-shoulder distance as the vertical distance between the knee and shoulder keypoints. Both references are expressed in image coordinates, so KHK and DKS are dimensionless percentages and do not depend on any metric measurement of the athlete. The athlete’s body height in centimeters is a manual input used only to convert pixel distances into metric units for the velocity-based characteristics, which are not considered in the present study.

All experiments ran on an Apple M4 Pro with 24 GB of unified memory. Configuration selection targeted variants that would fit on an entry-level dedicated GPU server marketed for AI inference: Hetzner’s GEX44, which contains an NVIDIA RTX 4000 SFF Ada with 20 GB of memory [42]. This server represents a realistic deployment environment for the automated system.

For each pose-estimation framework, the study selected the largest publicly available variant whose memory footprint and throughput suited this deployment class. The PyTorch-based configurations used PyTorch 2.1.2 with MPS; YOLO11x-Pose used Ultralytics 8.4.84, and RTMPose-L and ViTPose-L used MMPose 1.3.2 with MMDetection 3.2.0. MoveNet used TensorFlow 2.21.0. The non-maximum-suppression operator in the MMPose-based configurations lacks a Metal implementation and, therefore, ran on the CPU, while all neural-network inference remained on the GPU. The model checkpoint and input determine keypoint accuracy, so the accuracy results do not depend on the test hardware. By contrast, the throughput results reflect consumer Apple Silicon and provide relative rankings between configurations rather than absolute expectations for server-grade GPUs.

Each framework used a fixed, publicly available configuration. MoveNet used the SinglePose Thunder variant (v4, 256 × 256 input). YOLO11 used the largest pose variant, YOLO11x-Pose, with approximately 57M parameters and a 640-pixel input, trained on COCO. RTMPose used RTMPose-L at an input resolution of 384 × 288 pixels (MMPose configuration rtmpose-l_8xb256-420e_body8-384x288; checkpoint rtmpose-l_simcc-body7_pt-body7_420e-384x288). The checkpoint was trained on the Body7 collection, comprising COCO, AI Challenger, CrowdPose, MPII, sub-JHMDB, Halpe, and PoseTrack18, with the annotations converted to the 17-keypoint COCO format. The Body8 designation refers to evaluation on these datasets together with OCHuman rather than training on an eight-dataset collection [28,43]. ViTPose-L, with approximately 307 M parameters and a 256 × 192 input, was trained on COCO. Both top-down configurations, RTMPose-L and ViTPose-L, shared the same person detector: RTMDet-m, fine-tuned as a single-class person detector on COCO and Objects365 [44]. Both methods used the standard MMPose inference settings. In contrast, MoveNet and YOLO11x-Pose operated directly on the full video frame without the shared RTMDet-m person-detection and cropping stage.

### 3.2. Anomaly Detection

After pose estimation, the coordinates of each keypoint form a time series that describes the movement of the corresponding body part throughout the video. The pipeline must handle two error types: the model may assign a keypoint to the wrong limb, or it may place a keypoint away from the limb’s true position for an isolated frame. A third case, absent keypoints, requires no detection but must be handled before analysis. The correction stages run in a fixed order, each operating on the output of the previous one.

Limb-swap criterion: Pose estimators can exchange the left and right assignment of a limb, particularly when the legs cross or occlude one another. Such an error is invisible to per-trajectory outlier tests, because each individual time series remains smooth. The algorithm instead evaluates the two legs jointly. For each frame it compares the labeled assignment of the kicking and standing keypoints against the swapped assignment, measuring in each case the total displacement from the previously accepted positions. When the swapped assignment implies less motion, the frame is marked as swapped and the corresponding joints of both legs, that is heel, knee, and hip, are exchanged. The corrected positions then serve as the reference for the following frame, so that consecutive swapped frames are also detected. Because a single ankle may be absent while its limb is nonetheless swapped, the knee serves as secondary evidence and decides the comparison whenever the ankle pair cannot be evaluated.

Gap interpolation: Absent keypoints, including those left absent by a swap whose counterpart was missing, are filled by linear interpolation from the nearest valid values on either side. Interpolation is limited to gaps of at most three consecutive frames. Longer dropouts remain marked as missing rather than receiving a fabricated trajectory across frames in which the joint was never observed, and gaps at the start or end of a sequence, which lack a valid neighbor on one side, are never filled. Subsequent processing treats the remaining missing frames in the same way as frames with no detected keypoint.

Trajectory-excursion criterion: A correctly tracked keypoint is expected to move smoothly between neighboring frames. A single-frame mis-detection instead appears as a sudden sideways deviation from the surrounding trajectory. For each frame of the kicking leg’s heel, the algorithm, therefore, measures the perpendicular distance of the keypoint from the straight line connecting its positions in the preceding and following frames. If this distance exceeds 30% of the leg’s characteristic segment length, defined as the median projected heel–knee length, the frame is considered implausible. The heel position is then removed and replaced by linear interpolation from the surrounding valid frames. Because the criterion measures deviation perpendicular to the direction of movement, fast motion along the trajectory itself does not trigger the detector.

Videos that contain no implausible frames pass through all three stages unmodified. The numerical parameters of these criteria, namely the maximum interpolated gap of three frames and the cross-track threshold of 30% of the characteristic segment length, are heuristic values selected during development on the basis of visual inspection of the keypoint trajectories. Figure 1 presents examples of detected anomalies and their corrections.

The predefined node-detection rules require the angle of the athlete’s kicking leg. The system calculates these angles from the 2D coordinates of the kicking leg. Because the 2D analysis lacks depth information, the system estimates depth from the detected distances between the hip, knee, and ankle. It calculates and sorts the knee–hip and knee–ankle distances across the video, then uses the 97th percentile as an estimate of the true segment length (Figure 2).

The decision to use the 97th percentile was made during development based on the observation that one possible error of pose detection models is a false detection in which a limb is estimated to be excessively long. To reduce the influence of such erroneous detections on the analysis, a conservative approach was adopted by using the 97th percentile as the maximum limb length.

The system combines the estimated segment length with the detected 2D positions to approximate 3D vectors from the knee to the hip and from the knee to the ankle. The angle between these vectors provides the knee angle for each frame. The resulting knee-angle trajectory was then checked for anomalous values using a rule-based procedure similar to that applied to the keypoint coordinates.

To detect both MT nodes, the algorithm collects all frames with an angle below 110° and selects the candidate frame with the smallest *y* coordinate of the detected knee keypoint. This value represents the highest knee position as the image coordinates increase downward. To detect KE1, the algorithm searches after MT1 for the first sequence of three consecutively decreasing angle values and selects the first frame in that sequence.

For KE2, the system uses the vertical heel trajectory after MT2. It cleans, interpolates, and smooths the trajectory before the detection step. The algorithm then searches for the first sequence of three consecutive downward heel movements and selects the frame immediately before this sequence as KE2, because this frame represents the moment before the kicking leg begins to drop after the second kick. If the algorithm finds no valid heel-drop sequence, the system falls back to the original angle-based KE detection.

Parameters such as the 110° threshold for the MT node are direct technical translations of the predefined node definitions [12]. Other implementation-specific decisions were made based on observations during the iterative development process. Different detection methods were used for KE1 and KE2 after observing that some athletes lower the heel while flexing and extending the knee, whereas others raise the heel during knee flexion and extension for KE1. Similarly, the decision to smooth and interpolate the heel trajectory was made after observing that small inaccuracies in heel detection by the pose models could lead to incorrect node detection. In general, the implementation of the prototype was completed and finalized before the statistical analysis was conducted, in order to avoid retrospectively adapting technical details based on the statistical results. However, because the implementation was developed using 2D recordings that included, among others, recordings from the dataset subsequently evaluated, some degree of adaptation to the specific dataset may, therefore, have occurred.

Figure 3 shows five frames automatically detected from one particular performance of the double side kick.

### 3.3. Comparison of the Pose-Estimation Configurations

The following analyses compare the pose-estimation configurations in terms of their absolute errors, examine the effect of trajectory anomaly detection, and evaluate the agreement of the evaluated configurations with the 3D reference system.

#### 3.3.1. Primary Configuration Comparison

The primary statistical analysis compared the absolute errors of the configurations based on MoveNet, RTMPose-L, ViTPose-L, and YOLO11x-Pose within the final automated-analysis pipeline. This primary comparison was performed with anomaly detection disabled in order to evaluate differences between the pose-estimation configurations without the additional influence of trajectory anomaly correction. All four configurations processed all 27 kick executions. However, some executions yielded missing or invalid values for individual movement characteristics. The paired comparisons, therefore, excluded executions with incomplete data in the relevant characteristic group, as specified in the Section 2.

Table 3 summarizes the descriptive and inferential results of the primary configuration comparison. The Friedman test indicated a statistically significant configuration effect for phase-duration errors, $\chi^{2}\left(3\right)=11.73$, p=0.008, Kendall’s W=0.150, and femur-angle errors, $\chi^{2}\left(3\right)=13.08$, p=0.004, Kendall’s W=0.174. No statistically significant configuration effect was found for leg-spreading angles, $\chi^{2}\left(3\right)=4.83$, p=0.185, Kendall’s W=0.070, or distance- and height-based characteristics, $\chi^{2}\left(3\right)=6.60$, p=0.086, Kendall’s W=0.081.

For the phase-duration group, ViTPose-L produced the lowest median subject-level error. The post-hoc comparisons showed that ViTPose-L had significantly lower errors than YOLO11x-Pose after Holm correction, $p_{\mathrm{Holm}}=0.037$, with a matched-pairs rank-biserial correlation of $r_{\mathrm{rb}}=0.640$ in favor of ViTPose-L. No other pairwise comparison in the phase-duration group remained statistically significant after Holm correction.

For the femur-angle group, ViTPose-L again produced the lowest median error. The post-hoc comparisons showed that ViTPose-L had significantly lower errors than MoveNet after Holm correction, $p_{\mathrm{Holm}}=0.019$, with a matched-pairs rank-biserial correlation of $r_{\mathrm{rb}}=0.655$ in favor of ViTPose-L. No other pairwise comparison in the femur-angle group remained statistically significant after Holm correction.

The complete pairwise Wilcoxon signed-rank post-hoc results for the movement-characteristic groups with significant Friedman tests are provided in Appendix D.

For the leg-spreading-angle group, ViTPose-L showed the numerically lowest median descriptive error, while RTMPose-L showed the numerically lowest median descriptive error for the distance- and height-based characteristics. However, the Friedman tests for these two groups were not statistically significant, so no post-hoc comparisons were interpreted and these descriptive differences should not be understood as statistically supported model differences. Overall, the primary configuration comparison showed that pose-estimation configuration significantly affected phase-duration and femur-angle errors, but not all extracted movement-characteristic groups. The corresponding Kendall’s *W* values indicated small configuration effects.

#### 3.3.2. Input-Framing Sensitivity Analysis

A secondary sensitivity analysis evaluated whether the full-frame input used by MoveNet and YOLO11x-Pose influenced their relative performance. For this purpose, both models were additionally evaluated with the same RTMDet-m detector-crop procedure used by the top-down configurations. The analysis used complete paired observations for each full-frame and detector-crop comparison. Therefore, the descriptive medians in this subsection are pair-specific and may differ from the medians in Table 3, which were calculated on complete cases across the four primary configurations, and from the values shown in Figure 4.

For MoveNet, the detector-crop variant did not significantly reduce the errors in any movement-characteristic group after Holm correction. In the paired sensitivity subsets, the median errors changed from 0.056 to 0.064 s for phase durations (n=26), from 9.125° to 9.623° for femur angles (n=24), from 8.961° to 9.917° for leg-spreading angles (n=23), and from 7.830 to 6.811 percentage points for distance- and height-based characteristics (n=27).

For YOLO11x-Pose, detector cropping did not produce a consistent improvement. In the paired sensitivity subsets, the median phase-duration error changed from 0.072 to 0.067 s (n=26), and the distance- and height-based error changed from 7.308 to 9.460 percentage points (n=27). The angle errors increased with detector cropping, from 7.411° to 13.430° for femur angles (n=27) and from 7.856° to 13.966° for leg-spreading angles (n=27). The increase in leg-spreading-angle error remained statistically significant after Holm correction, W=64, $p_{\mathrm{raw}}=0.002$, $p_{\mathrm{Holm}}=0.015$, $r_{\mathrm{rb}}=0.661$, with the positive effect size indicating lower paired errors for the full-frame YOLO11x-Pose configuration.

Overall, the sensitivity analysis did not show that adding a detector crop consistently improved the full-frame configurations.

#### 3.3.3. Effect of Anomaly Detection

A secondary analysis evaluated the updated trajectory anomaly-detection module. Table 4 summarizes how often anomaly detection was activated and how often it affected the final evaluated movement characteristics. The detector was triggered at least once in 21 of 27 MoveNet analyses (77.8%), 9 of 27 RTMPose-L analyses (33.3%), 9 of 27 ViTPose-L analyses (33.3%), and 18 of 27 YOLO11x-Pose analyses (66.7%). However, not all anomaly triggers affected the final movement characteristics, because some triggers changed only auxiliary outputs such as kick velocity. When only the final evaluated characteristics were considered, anomaly detection changed the output in 14 MoveNet analyses (51.9%), 2 RTMPose-L analyses (7.4%), 3 ViTPose-L analyses (11.1%), and 10 YOLO11x-Pose analyses (37.0%). This shows that MoveNet and YOLO11x-Pose triggered the trajectory-correction procedure more frequently than RTMPose-L and ViTPose-L. As an exploratory analysis relevant to H2, the median absolute errors obtained with anomaly detection disabled were compared with the corresponding errors obtained with anomaly detection enabled. This comparison was performed separately for each pose-estimation configuration and each movement-characteristic group using paired Wilcoxon signed-rank tests. The resulting *p*-values were adjusted using the Holm correction across the 16 anomaly-detection comparisons. The full results of these tests are provided in Table A7 in Appendix C. Across the 16 comparisons, no test remained statistically significant after Holm correction. Given the small number of informative pairs in several comparisons, these results do not allow a conclusive evaluation of H2.

#### 3.3.4. Agreement with the 3D Reference System

The Bland–Altman agreement analysis used the anomaly-detection-enabled outputs and was performed for all four evaluated configurations. The full numerical Bland–Altman results are provided in Appendix A, with detailed results for ViTPose-L and RTMPose-L in Table A1 and the corresponding results for MoveNet and YOLO11x-Pose in Table A3.

The Shapiro–Wilk tests rejected normality of the paired differences for 7 of 17 characteristics for MoveNet, 9 of 17 characteristics for YOLO11x-Pose, 8 of 17 characteristics for ViTPose-L, and 11 of 17 characteristics for RTMPose-L. Following the Bland–Altman recommendation to consider transformation when the distributional assumptions of the differences are questionable [45], a log-transformed Bland–Altman check was calculated after log-transforming both the video-based and 3D reference values. This did not consistently resolve the normality violations. Normality was still rejected for 7 of 17 characteristics for MoveNet, 7 of 17 characteristics for YOLO11x-Pose, 9 of 17 characteristics for ViTPose-L, and 12 of 17 characteristics for RTMPose-L.

Because Bland and Altman also discuss non-parametric approaches when the assumptions of the conventional limits of agreement are questionable [45], a percentile-based sensitivity analysis was performed for model-characteristic combinations with non-normally distributed paired differences. This analysis used the empirical 2.5th and 97.5th percentiles of the paired differences instead of the conventional mean difference ±1.96 standard deviations. The full percentile-based sensitivity results are provided in Appendix A. The sensitivity analysis included 35 model-characteristic combinations with non-normally distributed paired differences. For 33 of these 35 combinations, the acceptable-agreement classification was unchanged compared with the conventional Bland–Altman limits. The only classification changes occurred for RTMPose-L: DKS1 changed from within to outside the predefined study-specific acceptable limit, whereas the leg-spreading angle at MT1 changed from outside to within the predefined study-specific acceptable limit. The overall number of characteristics classified as within the predefined study-specific acceptable limits, therefore, did not change.

Using the conventional Bland–Altman limits, MoveNet achieved acceptable agreement according to the predefined study-specific criteria for 3 of the 17 analyzed characteristics: DKS1, KHK1, and KI1 knee height. YOLO11x-Pose achieved acceptable agreement for one characteristic: DKS1. ViTPose-L achieved acceptable agreement for three characteristics: CH2, DKS1, and KI1 knee height. RTMPose-L also achieved acceptable agreement for three characteristics: CH2, the femur angle at MT1, and DKS1.

For all four configurations, the mean differences were generally small for many characteristics, indicating limited systematic bias. However, the 95% limits of agreement exceeded the predefined study-specific acceptable limits for most variables. This shows that the configurations can approximate selected characteristics well on average, but individual-level deviations from the 3D reference system remain substantial.

Overall, the Bland–Altman analysis showed that none of the evaluated configurations achieved acceptable agreement across the full set of extracted movement characteristics according to the predefined study-specific criteria. The percentile-based sensitivity analysis supported this interpretation, indicating that the main conclusion was not dependent on the conventional parametric limit-of-agreement calculation. Therefore, the automated 2D analysis is suitable for approximating selected movement characteristics, but it cannot fully replace 3D motion-capture analysis for precise individual-level assessment.

### 3.4. Performance Benchmark Results

Table 5 summarizes the performance benchmark. The MoveNet configuration achieved the highest end-to-end throughput, with a mean processing speed of 66.87 FPS across repetitions. YOLO11x-Pose ranked second at 23.57 FPS. RTMPose-L and ViTPose-L were substantially slower, with mean end-to-end processing speeds of 11.38 FPS and 6.50 FPS, respectively.

The pose-estimation-stage measurements produced the same ranking. MoveNet achieved the highest throughput at 79.29 FPS, followed by YOLO11x-Pose at 24.97 FPS. RTMPose-L reached 11.81 FPS, while ViTPose-L achieved the lowest throughput at 6.64 FPS. MoveNet showed the largest difference between end-to-end and pose-estimation-stage FPS, indicating that video decoding and per-frame processing overhead had a greater relative influence on the fastest configuration. For the slower configurations, the closer end-to-end and pose-estimation-stage values indicate that pose estimation dominated the total runtime.

The benchmark also recorded the percentage of frames missing at least one task-relevant keypoint. RTMPose-L produced no incomplete task-relevant frames in the benchmark summary. ViTPose-L also achieved very high detection completeness, with only 0.04% incomplete frames, compared with 0.63% for YOLO11x-Pose and 2.58% for MoveNet. Thus, the fastest configuration also produced the highest proportion of incomplete task-relevant detections, whereas the MMPose-based configurations provided the most complete keypoint availability.

Overall, the benchmark revealed a clear trade-off between runtime performance and keypoint completeness. The MoveNet configuration was by far the fastest and was, therefore, suited to high-throughput processing, but it produced the highest percentage of incomplete task-relevant frames. RTMPose-L and ViTPose-L provided the most complete task-relevant detections at substantially lower processing speeds. YOLO11x-Pose showed considerably higher throughput than RTMPose-L and ViTPose-L but lower detection completeness.

## 4. Discussion

The following sections address the four research questions in turn: the effect of model choice on accuracy, the agreement of the evaluated configurations with the reference system, the contribution of trajectory anomaly detection, and the runtime and completeness behavior that determines practical suitability.

### 4.1. Hypothesis 1: Effect of the Pose-Estimation Configuration

ViTPose-L performed most consistently across the characteristic groups, while RTMPose-L achieved similarly low errors for several groups, particularly phase durations and distance- and height-based characteristics. The statistical analysis identified significant configuration effects for phase durations and femur angles: the ViTPose-L configuration produced significantly lower phase-duration errors than YOLO11x-Pose and significantly lower femur-angle errors than MoveNet after Holm correction. Hypothesis 1 was, therefore, supported for these two characteristic groups. By contrast, no significant configuration effect was detected for leg-spreading angles or distance- and height-based characteristics, and Hypothesis 1 was not supported for these groups. Because the invalid outputs that led to exclusions from the complete-case comparison occurred for MoveNet, its reported errors are based only on executions for which it produced valid values. The comparison may, therefore, favor MoveNet by not reflecting these unsuccessful extractions.

The four configurations differ in how much of the image is devoted to the athlete. RTMPose-L [28] and ViTPose-L [41] operate top-down: the RTMDet-m person detection [44] is cropped from the frame and resampled to the network input, so the athlete fills that input. MoveNet [39] and YOLO11x-Pose [40,46] received the full frame, so the resolution available at the keypoints of interest scales with the fraction of the frame the athlete occupies. Nominal input size does not track this quantity: ViTPose-L takes a smaller input than MoveNet (256 × 192 against 256 × 256) but places all of it on the athlete.

This split coincides with the observed error ranking, but the sensitivity analysis did not show that detector cropping consistently improved the performance of MoveNet or YOLO11x-Pose. Input framing, therefore, does not appear to explain the observed differences on its own. The configurations also differ in capacity and training corpus, with RTMPose-L trained on the Body7 collection and the remaining configurations primarily trained on COCO [29]. Training corpus alone does not account for the ranking either, since MoveNet is additionally trained on yoga, fitness, and dance imagery selected for challenging poses and motion blur [39]. The observed differences, therefore, cannot be attributed to a single aspect of the evaluated configurations.

### 4.2. Benchmark Performance and Downstream Validity

A notable finding is that reported accuracy on the COCO benchmark [28,40,41] did not consistently correspond to performance in the downstream analysis. Recording quality, lighting conditions, and high-speed athlete movement may explain this discrepancy. Regardless of the exact cause, pose-estimation models can achieve similar scores on a benchmark such as COCO [29] yet perform significantly differently in a specific downstream task. Conventional pose-estimation benchmarks also do not directly evaluate the biomechanical accuracy of the resulting keypoints [47]. Benchmark accuracy alone may, therefore, be insufficient to guide the selection of a pose-estimation configuration for a specialized real-world system.

One explanation lies in the training data. A review of markerless motion capture for clinical gait biomechanics concludes that the open-source pose-estimation algorithms in current use “were never designed for biomechanical applications,” and identifies the labeling quality of their training datasets as a specific barrier to biomechanical use [48]. A benchmark score, therefore, reflects agreement with the corresponding pose annotations, which do not necessarily represent anatomically precise joint centers. Direct comparisons of monocular models against a marker-based reference show a wide spread in downstream error, with mean absolute errors between roughly 9° and 22° for knee flexion across eleven models [21]. The present results show a comparable spread within a single fixed pipeline, and extend it to characteristics that are not joint angles at all, such as the relative phase durations.

### 4.3. Agreement with the 3D Reference

The Bland–Altman analyses showed that low median absolute errors do not necessarily indicate sufficient individual-level agreement with the 3D reference. Bland–Altman analysis evaluates agreement between two measurement methods rather than only their correlation or average error [36]. Using the conventional Bland–Altman limits of agreement, across the four evaluated configurations with anomaly detection enabled, the mean differences were generally small for many characteristics, indicating limited systematic bias. However, the 95% limits of agreement exceeded the predefined study-specific acceptable limits for most variables. Because several paired differences were non-normally distributed, a log-transformed Bland–Altman check and a percentile-based sensitivity analysis were additionally performed. The log transformation did not consistently resolve the normality violations, and the percentile-based sensitivity analysis changed the acceptable-agreement classification for only 2 of the 35 non-normal model-characteristic combinations. The overall number of characteristics classified as within the predefined study-specific acceptable limits remained unchanged. MoveNet met these limits for 3 of the 17 characteristics: DKS1, KHK1, and KI1 knee height. YOLO11x-Pose met them for one characteristic: DKS1. ViTPose-L met them for three characteristics: CH2, DKS1, and KI1 knee height. RTMPose-L also met them for three characteristics: CH2, the femur angle at MT1, and DKS1. Thus, the evaluated configurations can approximate a small subset of characteristics with acceptable agreement according to the predefined study-specific criteria, but most characteristics still showed limits of agreement that were too wide for precise biomechanical measurement of individual kick executions.

The distinction between systematic bias and limits of agreement directly affects the system’s practical use. Small mean differences indicate that the video-based analysis does not consistently overestimate or underestimate most characteristics by a large amount. Wide limits of agreement, however, indicate insufficient reliability for some characteristics at the individual level. This limitation is particularly important when the system provides feedback to individual athletes because a large error in a single execution could lead to a misleading interpretation.

Using Bland–Altman analysis to compare a 2D markerless pipeline with 3D motion capture during a floor-to-waist lifting test, mean differences of 1.9° to 22.1° were reported for posture and coordination measures, with limits of agreement of approximately ±20° for most measures, leading the authors to conclude that the approach is usable for objective movement measurement but that its agreement with 3D capture is at present insufficient [18]. That conclusion, reached for a controlled sagittal-plane lifting task, closely matches the one supported by the present data for a considerably more demanding technique. Other task-specific studies report comparable magnitudes. Sagittal-plane hip, knee, and ankle angles during gait were estimated with mean absolute errors of 4.0°, 5.6°, and 7.4° [49]. A markerless multi-camera application evaluated across five athletic tasks achieved its lowest error on the squat, with a mean RMSE of 7.0° across joint angles and a range of 2.9° to 13.6° within that task alone; the more dynamic jumping, hopping, and cutting tasks performed less well [50]. For single-camera markerless analysis of baseball pitching, time-series RMSE ranged from 4.37° for trunk lateral tilt to 20.78° for glove-arm shoulder external rotation, with the trunk and pelvis measures the most accurate of the set [19]. Two observations follow. First, errors of this magnitude are the norm rather than the exception for markerless pipelines validated against a marker-based reference. Second, the error is strongly variable-dependent within a single study, which mirrors the pattern observed here across the characteristic groups.

### 4.4. Angle Characteristics and Camera Constraints

The angle-based characteristics remain the most difficult to estimate. Although ViTPose-L achieved the lowest median errors for the femur-angle and leg-spreading-angle groups in the primary comparison, the Bland–Altman limits of agreement exceeded the predefined range for most angle variables. The single 2D camera view likely contributes substantially to this limitation. Previous studies show that 2D movement analysis can provide useful kinematic information [16], but the recording setup, camera perspective, movement plane, and amount of out-of-plane motion strongly affect its accuracy [17,37]. Even improved pose estimation cannot recover the movement’s full depth component from image-plane keypoints. The pseudo-3D geometric calculation is intended to mitigate this limitation but cannot fully reconstruct the 3D orientation of the limbs. Consequently, a single camera cannot accurately estimate all angle characteristics affected by out-of-plane motion.

The frame rate of the source videos also affects the phase-duration results. At 24 fps, one frame corresponds to approximately 0.042 s. For short phases, an error of only one or two frames can noticeably change the calculated duration. This limitation is especially relevant for KI1 because its predefined study-specific acceptable limit is close to the duration of a single frame. Higher frame rates would improve temporal resolution and could enable more precise detection of short phases.

An investigation of automated 2D motion analysis in running concluded that a minimum video sampling rate of 100 Hz is required for reliable event detection. The study reported that misidentifying the moment of initial foot contact by only 20 ms produced differences of up to 20° in the knee compression angle, defined as the change in sagittal-plane knee angle between initial foot contact and foot flat. This occurred even though the detected keypoints themselves agreed well with the reference system [51]. Since the present videos were recorded at 24 fps, a single-frame error corresponds to approximately 42 ms and can, therefore, have a substantial effect on short phase durations and angle values at specific movement nodes. In comparison, 2D validation studies reporting good agreement for continuous kinematics used substantially higher sampling rates, such as 120 Hz [16].

The placement of the camera is also relevant as joint angles estimated from four different viewing angles can differ significantly from one another [37].

### 4.5. Hypothesis 2: Effect of Trajectory Anomaly Detection

The anomaly detector identified irregularities more often for MoveNet and YOLO11x-Pose than for RTMPose-L and ViTPose-L. It triggered in 77.8% of MoveNet analyses and 66.7% of YOLO11x-Pose analyses, compared with 33.3% for both RTMPose-L and ViTPose-L. The difference is larger still for the triggers that actually propagated to the reported characteristics: 51.9% of MoveNet and 37.0% of YOLO11x-Pose analyses, against 7.4% for RTMPose-L and 11.1% for ViTPose-L. Nevertheless, enabling anomaly detection did not significantly reduce the final movement-characteristic errors after correction for multiple comparisons. Because several comparisons were based on only one to three informative pairs, Hypothesis 2 could not be evaluated conclusively with the available data. The results should, therefore, be interpreted descriptively. For RTMPose-L and ViTPose-L, the detector altered the reported characteristics in at most 3 of the 27 analyses. Because zero-difference pairs were excluded from the Wilcoxon signed-rank tests, several comparisons were based on only one to three informative pairs, which limits the confirmatory value of the anomaly-detection analysis.

For MoveNet and YOLO11x-Pose, anomaly detection affected more analyses, but the changes did not occur in a consistent direction. The median absolute error increased in two of the 16 comparisons, by (0.59°) for the MoveNet femur angles and by (2.06°) for the YOLO11x-Pose leg-spreading angles. The anomaly-detection module should, therefore, be regarded primarily as a robustness and diagnostic component rather than as a procedure that systematically improves measurement accuracy. A further consideration is that only the frames selected as technique nodes contribute directly to the reported movement characteristics. An anomaly detected elsewhere in the sequence may, therefore, be corrected without affecting any of the final evaluated values.

The gap-interpolation stage follows established practice: Sports2D likewise interpolates missing pose and angle sequences only where the gaps are not too large, before filtering them [52]. However, no smoothing filter was applied at any stage. This was intentional, because the analysis reads keypoint positions at a small number of predefined frames rather than describing how keypoints behave across the whole video, and smoothing was judged to risk altering the values read at those frames.

### 4.6. Performance Benchmark

The performance benchmark revealed a clear trade-off between runtime and keypoint completeness in the tested hardware and software environment. MoveNet achieved the highest throughput by a large margin, with a mean end-to-end processing speed of 66.87 FPS, but also produced the highest proportion of incomplete task-relevant keypoint frames. YOLO11x-Pose achieved the second-highest throughput at 23.57 FPS and occupied an intermediate position for keypoint completeness. RTMPose-L and ViTPose-L were substantially slower, with mean end-to-end speeds of 11.38 FPS and 6.50 FPS, but produced the most complete task-relevant detections. Similar research evaluated many of the same pose-estimation configurations and reported a comparable trade-off between accuracy and analysis speed [53].

The measured throughput is considerably lower than inference speeds reported for the underlying pose-estimation models in isolation; RTMPose-L has been reported at more than 130 FPS under optimized GPU inference [28]. The difference is expected and reflects what is being measured. The values reported here are end-to-end and include video decoding, person detection where applicable, keypoint inference, trajectory assembly, anomaly detection, and characteristic extraction, whereas published benchmarks typically report inference latency alone under an optimized runtime.

Furthermore, the benchmark was conducted on an Apple M4 Pro using framework-specific execution backends, whereas the intended deployment environment uses an NVIDIA GPU. The observed runtime ranking should, therefore, be interpreted as specific to the tested environment, as relative performance may change across hardware platforms, framework backends, and optimized inference runtimes. Benchmarking the complete configurations on the intended deployment hardware is required before making deployment-specific runtime recommendations.

### 4.7. Limitations and Future Work

The main limitations of the present study are the single-camera recording setup, the low frame rate of the available videos, and the limited sample size of 27 kick executions. The single 2D camera view restricts the estimation of depth-dependent angle characteristics, while the 24 fps recording rate limits the precision of short temporal characteristics such as KI1, for which one frame corresponds to 41.7 ms. In addition, the anomaly-detection analysis had limited confirmatory value because several comparisons contained only one to three informative non-zero pairs.

A further limitation is that the predefined agreement limits for the duration, distance-, and height-based characteristics were exploratory and sample-dependent. They should, therefore, be interpreted as study-specific criteria for approximate feedback rather than as generally applicable biomechanical thresholds.

The present study also does not represent a fully independent external validation. Some heuristic parameters and implementation decisions were developed using recordings from the same dataset that was subsequently used for evaluation. Although the final analysis was applied consistently across all evaluated pose-estimation configurations, this may have introduced dataset-specific optimization and should be considered when interpreting the results.

Several directions follow from these limitations. A second camera view would address the missing depth component directly, which the present results suggest is the main limitation for the angle characteristics [54,55]. Approaches also exist to enrich pose estimation with biomechanical information, for example by predicting a denser, anatomically defined set of keypoints [47]. Finally, the ranking of the evaluated pose-estimation configurations should be re-tested on higher-frame-rate recordings and under varied camera positions and lighting. Future validation should also be performed on an independent external dataset that was not used during parameter selection or system development.

## 5. Conclusions

The findings demonstrate that an automated system can extract predefined movement characteristics from 2D videos of the double side kick. However, the Bland–Altman results show that the predefined study-specific acceptable limits were met only for a small subset of the 17 evaluated characteristics. The system should, therefore, be interpreted as a practical tool for approximate feedback on selected characteristics rather than as a replacement for 3D motion capture or as a source of precise individual-level biomechanical measurement.

The choice of pose-estimation configuration measurably affected selected components of the analysis. Configuration choice had a statistically significant effect on the phase-duration and femur-angle characteristics, whereas no statistically significant configuration effect was detected for the leg-spreading angles or the distance- and height-based characteristics. ViTPose-L and RTMPose-L showed the most favorable overall accuracy and completeness, while MoveNet offered the highest runtime performance in the tested hardware and software environment. Reported benchmark accuracy did not consistently correspond to downstream performance, so configuration selection still requires validation on the target task.

Future work should investigate whether higher frame rates, multi-camera setups, or alternative pose-estimation configurations can further improve agreement with the 3D reference measurements.

## Figures and Tables

**Figure 1:**
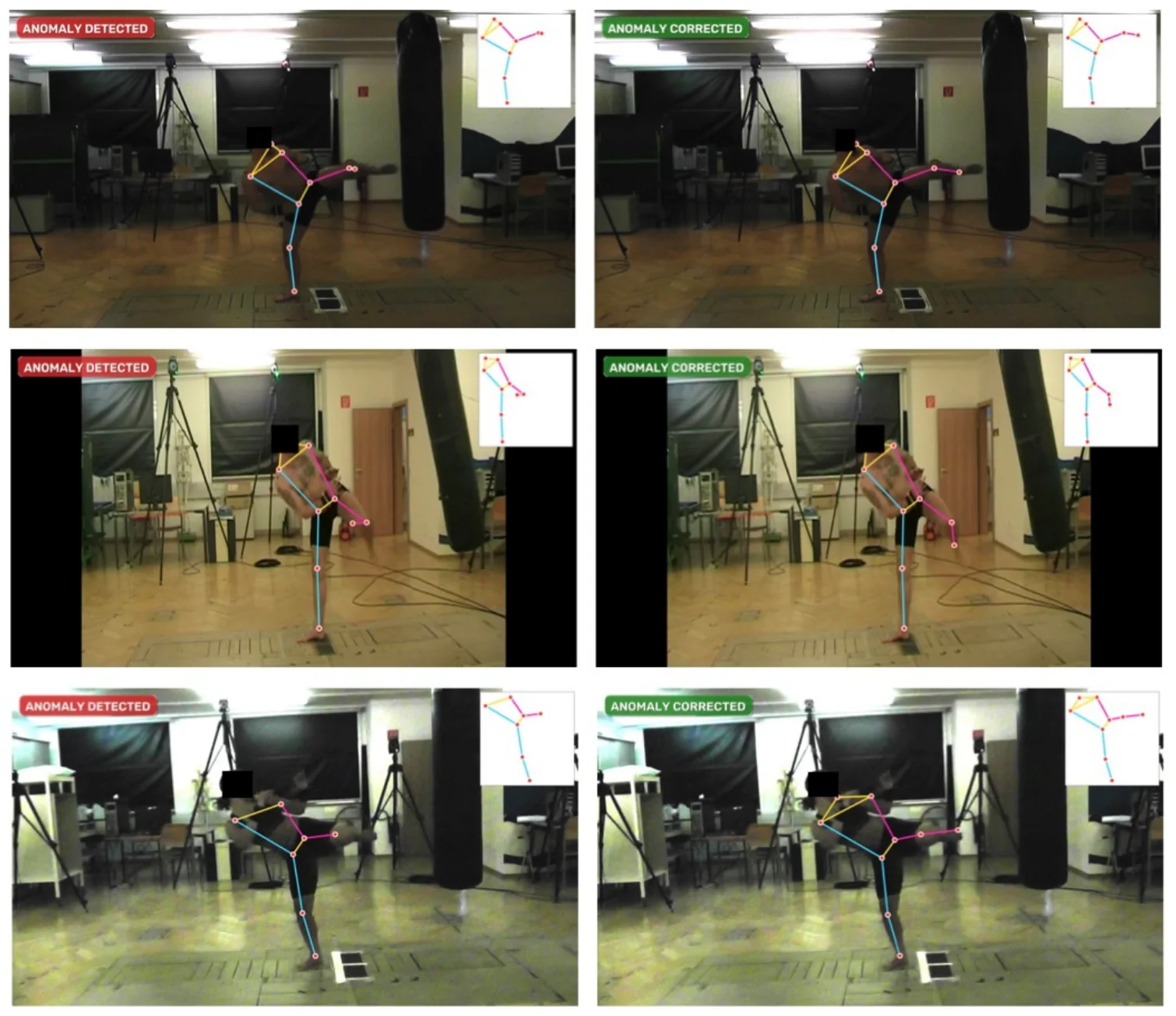
Examples of detected keypoint anomalies and their corrections. Each panel shows an anomalous keypoint trajectory identified by the limb-swap or trajectory-excursion criterion, together with the corrected trajectory after exchanging the affected joints of the two legs or after removing the implausible frame and replacing it by linear interpolation from the surrounding valid frames.

**Figure 2:**
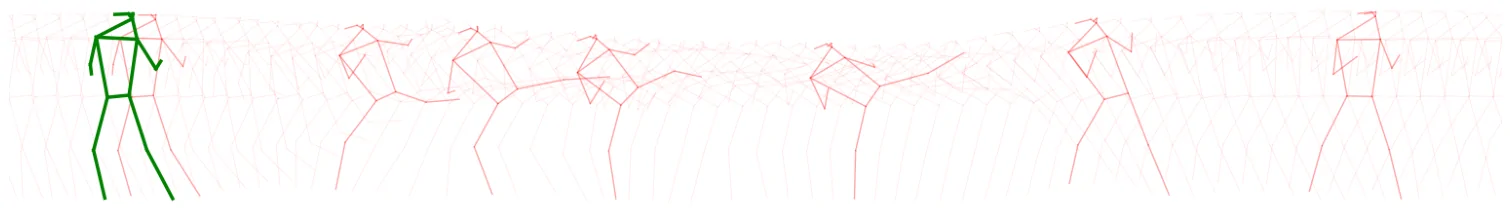
Full-body kinogram of the technique execution. The figure arranges all detected poses in temporal order and offsets them horizontally for readability. It emphasizes every tenth pose and highlights in green the pose corresponding to the 97th percentile of the projected kicking-leg length.

**Figure 3:**
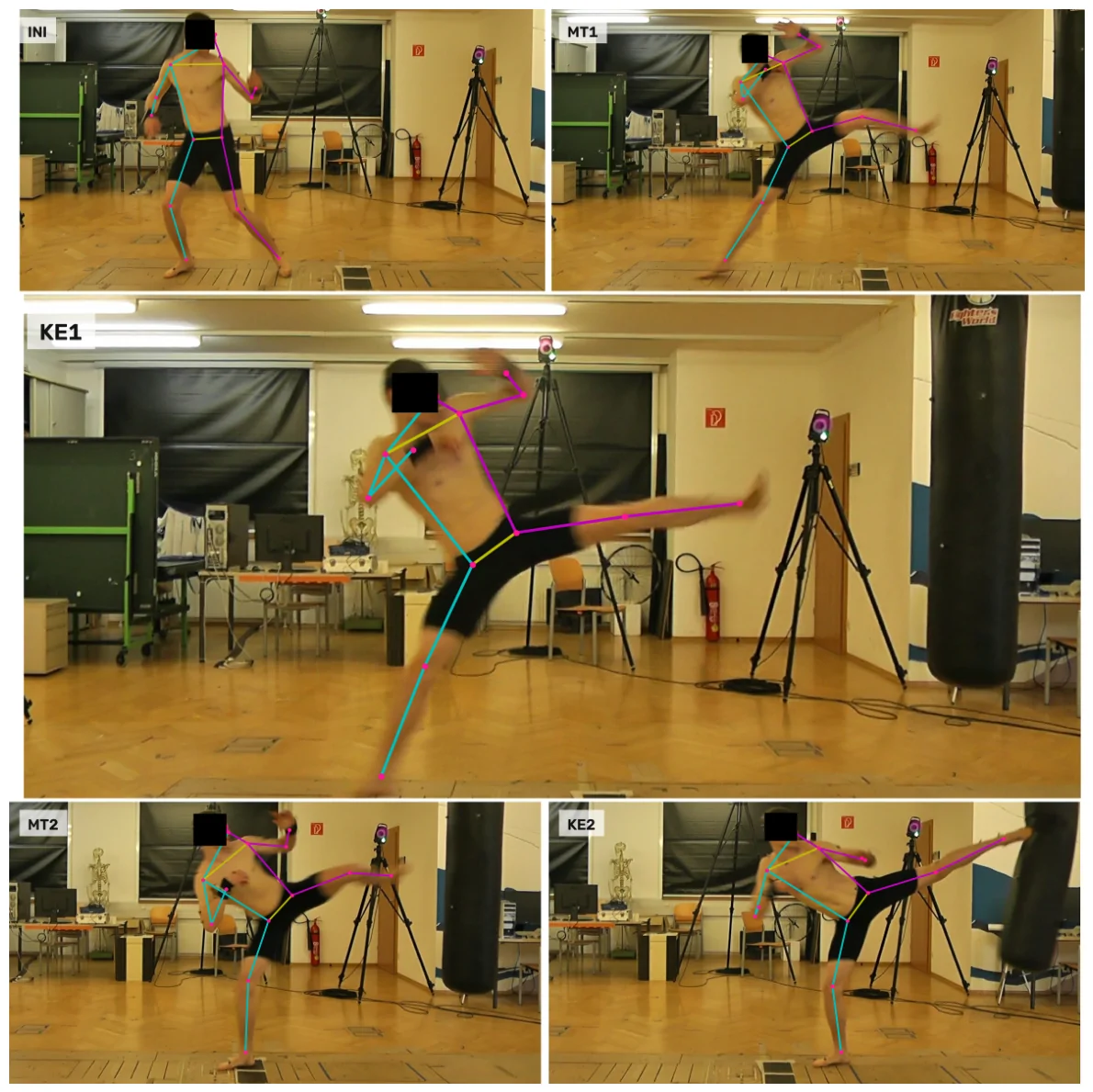
Detected movement nodes used for the automated analysis of the double side kick execution. The panels show the initial position (INI), first chamber position (MT1), first kick impact position (KE1), second chamber position (MT2), and second kick impact position (KE2).

**Figure 4:**
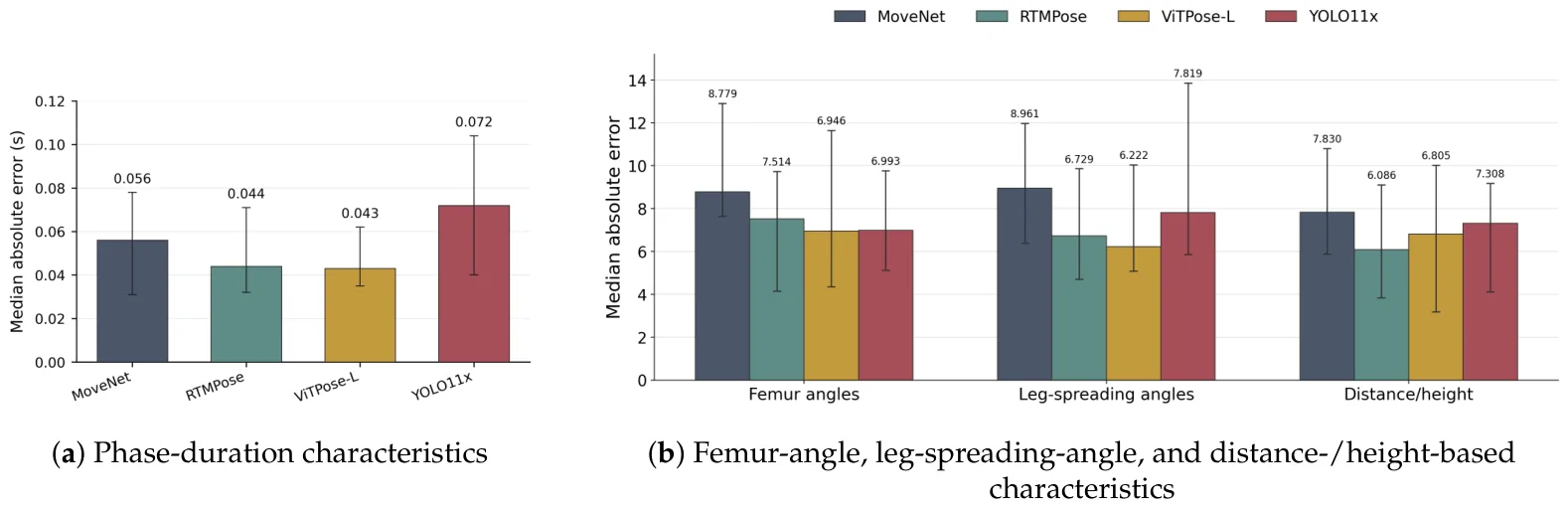
Median absolute errors by pose-estimation model across the movement-characteristic groups. Error bars represent the interquartile range (IQR). Lower values indicate better agreement with the 3D reference. Panel (**a**) shows the phase-duration characteristics separately due to the smaller error scale. Panel (**b**) shows the remaining groups.

**Table 1:** Performance indicators extracted from the double side kick [12,23].

Characteristic Group	Variables	Description
Phase durations	CH1, KI1, CH2, KI2	Duration of the four movement phases of the double side kick.
Distances	KHK, DKS	KHK represents the kicking leg’s knee height normalized by the trochanter major height in a neutral standing position and expressed as a percentage. DKS represents the distance between the kicking knee and the nearest shoulder normalized by the same distance in a neutral standing position and expressed as a percentage. Both measures are relative percentage values.
Vector spreading angle (VSA)	Femur vector angle and residual leg vector angle	The femur angle measures the angle between the vectors connecting each hip center of rotation to the corresponding knee point. The leg angle measures the angle between the vectors connecting each hip center of rotation to the corresponding ankle center of rotation.

**Table 2:** Nodes and phases of the double side kick used for the analysis [12].

Type	Name	Description
Node 1	Initialization (INI)	Moment when the kicking leg’s foot loses contact with the ground.
Phase 1	Chambering 1 (CH1)	First preparation phase of the kick, consisting mainly of knee flexion as well as hip flexion and abduction of the kicking leg.
Node 2	Time of measurement 1 (MT1)	Highest elevation of the kicking leg’s knee before the knee angle of the kicking leg surpasses 110°.
Phase 2	Kicking phase 1 (KI1)	First kicking phase, consisting mainly of extension of the knee and hip joints of the kicking leg.
Node 3	Knee extension maximum 1 (KE1)	End of the first kick, defined by the maximum extension of the kicking leg’s knee.
Phase 3	Chambering 2 (CH2)	Re-chambering phase and preparation for the second kick.
Node 4	Time of measurement 2 (MT2)	Highest elevation of the kicking leg’s knee after the first kick and before the second kicking phase.
Phase 4	Kicking phase 2 (KI2)	Second kicking phase, defined in the same way as the first kicking phase.
Node 5	Knee extension maximum 2 (KE2)	Maximum extension of the kicking leg’s knee and hip during the second kick, leading to target contact.

**Table 3:** Summary of the primary model comparison for each movement-characteristic group. Values represent median subject-level absolute errors with interquartile ranges shown in parentheses. Values are reported in seconds for phase durations, degrees for angle characteristics, and percentage points for distance- and height-based characteristics. The lowest median error in each row is shown in bold.

Movement-Characteristic Group	*n*	MoveNet	RTMPose-L	ViTPose-L	YOLO11x	Friedman *p*	*W*
Phase durations	26	0.056 (0.031–0.078)	0.044 (0.032–0.071)	**0.043** **(0.035–0.062)**	0.072 (0.040–0.104)	0.008	0.150
Femur angles	25	8.779 (7.637–12.909)	7.514 (4.148–9.726)	**6.946** **(4.353–11.648)**	6.993 (5.128–9.760)	0.004	0.174
Leg-spreading angles	23	8.961 (6.386–11.972)	6.729 (4.712–9.860)	**6.222** **(5.083–10.038)**	7.819 (5.866–13.850)	0.185	0.070
Distance- and height-based characteristics	27	7.830 (5.883–10.806)	**6.086** **(3.843–9.102)**	6.805 (3.194–10.023)	7.308 (4.122–9.181)	0.086	0.081

**Table 4:** Summary of anomaly detection activity for each pose-estimation model.

Pose-Estimation Model	Any Anomaly Trigger	Final Characteristics Changed	Changed Indices	Changed Final-Characteristic Entries
MoveNet	21/27 (77.8%)	14/27 (51.9%)	19	120
RTMPose-L	9/27 (33.3%)	2/27 (7.4%)	1	14
ViTPose-L	9/27 (33.3%)	3/27 (11.1%)	3	28
YOLO11x-Pose	18/27 (66.7%)	10/27 (37.0%)	16	85

**Table 5:** Performance benchmark results for the evaluated pose-estimation models. Incomplete task-relevant frames are reported as raw frame counts and percentages of all timed frames.

Pose-Estimation Model	Runs	Timed Videos	End-to-End FPS	Inference FPS	Incomplete Task-Relevant Frames
MoveNet	5	135	66.87 ± 0.36	79.29	350/13,560 (2.58%)
RTMPose-L	5	135	11.38 ± 0.09	11.81	0/13,560 (0.00%)
ViTPose-L	5	135	6.50 ± 0.10	6.64	5/13,560 (0.04%)
YOLO11x-Pose	5	135	23.57 ± 0.05	24.97	85/13,560 (0.63%)

## Data Availability

The source video recordings and the corresponding 3D motion-capture reference data are not publicly available, because they contain identifiable footage of the participants and their use is governed by the consent obtained for the original data collection [12]. The aggregated per-model error values, the benchmark measurements underlying Table 3, Table 4 and Table 5, and the analysis code are available from the corresponding author on reasonable request.

## Abbreviations

The following abbreviations are used in this manuscript:

2D	Two-dimensional
3D	Three-dimensional
FPS	Frames per second
LoA	Limits of agreement
INI	Initialization, node 1 of the double side kick
CH1	Chambering 1, first preparation phase
MT1	Time of measurement 1, node 2
KI1	Kicking phase 1
KE1	Knee extension maximum 1, node 3
CH2	Chambering 2, re-chambering phase
MT2	Time of measurement 2, node 4
KI2	Kicking phase 2
KE2	Knee extension maximum 2, node 5
KHK	Knee height of the kicking leg, normalized by trochanter major height
DKS	Distance between the kicking knee and the nearest shoulder
VSA	Vector spreading angle
COCO	Common Objects in Context, keypoint dataset and 17-keypoint format
CPU	Central processing unit
GPU	Graphics processing unit
MPS	Metal Performance Shaders
NMS	Non-maximum suppression
IMU	Inertial measurement unit
RMSE	Root mean square error

## Appendix A. Detailed Bland–Altman Results

**Table A1:** Detailed Bland–Altman results for ViTPose-L and RTMPose-L with anomaly detection enabled. Differences were calculated as video-based value minus 3D reference value. LoA = limits of agreement.

Characteristic	ViTPose-L		RTMPose-L		Acceptable LoA	Within Limit
	Mean Diff.	95% LoA	Mean Diff.	95% LoA		
Phase durations, s						
CH1	0.032	[−0.166; 0.230]	0.031	[−0.231; 0.292]	±0.162	–
KI1	0.027	[−0.083; 0.136]	0.022	[−0.059; 0.104]	±0.050	–
CH2	0.047	[−0.063; 0.158]	0.050	[−0.065; 0.166]	±0.190	Both
KI2	−0.005	[−0.137; 0.127]	−0.028	[−0.172; 0.116]	±0.108	–
Femur angles, °						
MT1	1.25	[−20.87; 23.37]	−0.61	[−19.60; 18.38]	±20	RTMPose-L
KI1/KE1	4.04	[−15.94; 24.01]	0.19	[−22.62; 23.00]	±20	–
MT2	2.26	[−23.90; 28.42]	0.54	[−31.77; 32.85]	±20	–
KI2/KE2	−5.09	[−26.35; 16.18]	−7.64	[−42.05; 26.77]	±20	–
Leg-spreading angles, °						
MT1	5.69	[−13.61; 24.99]	4.76	[−10.64; 20.16]	±20	–
KI1/KE1	6.78	[−13.99; 27.55]	4.90	[−13.85; 23.65]	±20	–
MT2	1.18	[−18.97; 21.32]	0.39	[−26.75; 27.52]	±20	–
KI2/KE2	1.25	[−19.60; 22.11]	−2.48	[−34.88; 29.93]	±20	–
Distance- and height-based characteristics, percentage points						
DKS1	2.02	[−7.31; 11.34]	0.33	[−13.93; 14.59]	±15.5	Both
DKS2	2.76	[−16.11; 21.64]	4.12	[−14.89; 23.13]	±20.0	–
KHK1	−2.19	[−25.94; 21.56]	−2.68	[−30.87; 25.51]	±22.0	–
KI1 knee height	−0.70	[−27.10; 25.69]	−3.47	[−36.88; 29.94]	±28.5	ViTPose-L
KHK2	1.23	[−28.68; 31.15]	−6.15	[−45.65; 33.35]	±16.5	–

**Table A2:** Ninety-five-percent confidence intervals for the mean differences and limits of agreement for ViTPose-L with anomaly detection enabled. Differences were calculated as video-based value minus 3D reference value. LoA = limits of agreement.

Characteristic	Mean Diff. 95% CI	Lower LoA 95% CI	Upper LoA 95% CI
Phase durations, s			
CH1	[−0.008; 0.073]	[−0.236; −0.095]	[0.160; 0.301]
KI1	[0.004; 0.049]	[−0.122; −0.044]	[0.097; 0.175]
CH2	[0.025; 0.070]	[−0.102; −0.024]	[0.118; 0.197]
KI2	[−0.032; 0.022]	[−0.184; −0.090]	[0.080; 0.174]
Femur angles, °			
MT1	[−3.21; 5.72]	[−28.60; −13.14]	[15.64; 31.11]
KI1/KE1	[0.00; 8.07]	[−22.92; −8.95]	[17.02; 30.99]
MT2	[−3.02; 7.54]	[−33.05; −14.76]	[19.28; 37.57]
KI2/KE2	[−9.38; −0.79]	[−33.78; −18.92]	[8.74; 23.61]
Leg-spreading angles, °			
MT1	[1.79; 9.58]	[−20.36; −6.86]	[18.24; 31.74]
KI1/KE1	[2.58; 10.97]	[−21.26; −6.73]	[20.29; 34.81]
MT2	[−2.89; 5.24]	[−26.01; −11.93]	[14.28; 28.36]
KI2/KE2	[−2.95; 5.46]	[−26.89; −12.31]	[14.82; 29.40]
Distance- and height-based characteristics, percentage points			
DKS1	[0.14; 3.90]	[−10.56; −4.05]	[8.08; 14.60]
DKS2	[−1.05; 6.57]	[−22.71; −9.51]	[15.04; 28.24]
KHK1	[−6.98; 2.60]	[−34.24; −17.64]	[13.26; 29.86]
KI1 knee height	[−6.03; 4.63]	[−36.32; −17.87]	[16.47; 34.92]
KHK2	[−4.80; 7.27]	[−39.14; −18.22]	[20.69; 41.61]

**Table A3:** Detailed Bland–Altman results for MoveNet and YOLO11x-Pose with anomaly detection enabled. Differences were calculated as video-based value minus 3D reference value. LoA = limits of agreement.

Characteristic	MoveNet		YOLO11x-Pose		Acceptable LoA	Within Limit
	Mean Diff.	95% LoA	Mean Diff.	95% LoA		
Phase durations, s						
CH1	0.043	[−0.161; 0.247]	0.003	[−0.217; 0.223]	±0.162	–
KI1	0.041	[−0.160; 0.241]	0.018	[−0.058; 0.093]	±0.050	–
CH2	0.031	[−0.152; 0.213]	0.177	[−0.215; 0.570]	±0.190	–
KI2	−0.011	[−0.231; 0.209]	−0.049	[−0.273; 0.174]	±0.108	–
Femur angles, °						
MT1	4.29	[−14.86; 23.44]	−1.58	[−21.01; 17.85]	±20	–
KI1/KE1	6.84	[−16.43; 30.11]	−0.31	[−23.68; 23.06]	±20	–
MT2	2.49	[−27.12; 32.11]	−5.93	[−42.26; 30.40]	±20	–
KI2/KE2	−5.95	[−58.26; 46.36]	−16.63	[−54.04; 20.78]	±20	–
Leg-spreading angles, °						
MT1	4.06	[−32.35; 40.47]	2.08	[−18.31; 22.46]	±20	–
KI1/KE1	7.23	[−15.24; 29.70]	2.20	[−18.88; 23.28]	±20	–
MT2	−3.62	[−38.22; 30.98]	−9.55	[−39.57; 20.46]	±20	–
KI2/KE2	−3.60	[−55.40; 48.20]	−14.67	[−51.12; 21.79]	±20	–
Distance- and height-based characteristics, percentage points						
DKS1	1.90	[−9.09; 12.89]	2.80	[−8.41; 14.00]	±15.5	Both
DKS2	1.54	[−17.92; 21.00]	5.88	[−12.39; 24.15]	±20.0	–
KHK1	−0.07	[−18.53; 18.39]	−2.63	[−33.71; 28.46]	±22.0	MoveNet
KI1 knee height	−0.66	[−28.35; 27.03]	−4.16	[−31.34; 23.02]	±28.5	MoveNet
KHK2	−6.17	[−47.58; 35.24]	−4.32	[−58.50; 49.85]	±16.5	–

**Table A4:** Percentile-based Bland–Altman sensitivity analysis for model-characteristic combinations with non-normally distributed paired differences. The table is restricted to combinations for which the Shapiro–Wilk test rejected normality; combinations without evidence of non-normality are reported only in the main Bland–Altman tables. Percentile-based limits were calculated as the empirical 2.5th and 97.5th percentiles of the paired differences and compared with the same predefined study-specific acceptable limits. LoA = limits of agreement.

Model	Characteristic	*n*	Percentile-Based LoA	Acceptable LoA	Parametric Within Limit	Percentile Within Limit
MoveNet	CH1	26	[−0.086; 0.279]	±0.162	–	–
MoveNet	KI1	26	[−0.051; 0.253]	±0.050	–	–
MoveNet	Femur KI2/KE2	25	[−66.57; 11.36]	±20	–	–
MoveNet	Leg-spreading MT1	27	[−34.99; 21.32]	±20	–	–
MoveNet	Leg-spreading MT2	27	[−47.26; 17.81]	±20	–	–
MoveNet	Leg-spreading KI2/KE2	26	[−69.51; 22.76]	±20	–	–
MoveNet	KHK2	27	[−57.86; 15.17]	±16.5	–	–
RTMPose-L	CH1	26	[−0.216; 0.352]	±0.162	–	–
RTMPose-L	CH2	26	[−0.019; 0.172]	±0.190	Within	Within
RTMPose-L	Femur MT2	27	[−39.33; 17.94]	±20	–	–
RTMPose-L	Femur KI2/KE2	27	[−56.15; 11.29]	±20	–	–
RTMPose-L	Leg-spreading MT1	27	[−11.33; 14.16]	±20	–	Within
RTMPose-L	Leg-spreading MT2	27	[−32.74; 17.81]	±20	–	–
RTMPose-L	Leg-spreading KI2/KE2	27	[−44.47; 19.60]	±20	–	–
RTMPose-L	DKS1	27	[−16.65; 8.82]	±15.5	Within	–
RTMPose-L	KHK1	27	[−29.19; 24.84]	±22.0	–	–
RTMPose-L	KI1 knee height	27	[−31.68; 37.63]	±28.5	–	–
RTMPose-L	KHK2	27	[−57.25; 12.68]	±16.5	–	–
ViTPose-L	CH1	26	[−0.110; 0.227]	±0.162	–	–
ViTPose-L	KI1	26	[−0.036; 0.149]	±0.050	–	–
ViTPose-L	CH2	26	[−0.016; 0.190]	±0.190	Within	Within
ViTPose-L	Femur MT1	27	[−25.55; 18.18]	±20	–	–
ViTPose-L	Femur KI2/KE2	27	[−30.62; 9.43]	±20	–	–
ViTPose-L	Leg-spreading KI1/KE1	27	[−6.37; 26.61]	±20	–	–
ViTPose-L	KHK1	27	[−28.69; 16.19]	±22.0	–	–
ViTPose-L	KHK2	27	[−35.67; 20.36]	±16.5	–	–
YOLO11x-Pose	KI1	26	[−0.032; 0.091]	±0.050	–	–
YOLO11x-Pose	CH2	26	[−0.071; 0.583]	±0.190	–	–
YOLO11x-Pose	KI2	26	[−0.174; 0.204]	±0.108	–	–
YOLO11x-Pose	Femur MT2	27	[−51.19; 19.36]	±20	–	–
YOLO11x-Pose	Femur KI2/KE2	27	[−62.51; 4.04]	±20	–	–
YOLO11x-Pose	Leg-spreading MT1	27	[−22.62; 19.83]	±20	–	–
YOLO11x-Pose	Leg-spreading KI2/KE2	27	[−52.22; 5.02]	±20	–	–
YOLO11x-Pose	KHK1	27	[−43.84; 20.27]	±22.0	–	–
YOLO11x-Pose	KHK2	27	[−57.95; 24.77]	±16.5	–	–

**Table A5:** Ninety-five-percent confidence intervals for the mean differences and limits of agreement for RTMPose-L with anomaly detection enabled. Differences were calculated as video-based value minus 3D reference value. LoA = limits of agreement.

Characteristic	Mean Diff. 95% CI	Lower LoA 95% CI	Upper LoA 95% CI
Phase durations, s			
CH1	[−0.023; 0.084]	[−0.324; −0.137]	[0.199; 0.385]
KI1	[0.006; 0.039]	[−0.088; −0.030]	[0.075; 0.133]
CH2	[0.027; 0.074]	[−0.106; −0.024]	[0.125; 0.207]
KI2	[−0.058; 0.002]	[−0.224; −0.121]	[0.065; 0.168]
Femur angles, °			
MT1	[−4.44; 3.22]	[−26.24; −12.96]	[11.74; 25.02]
KI1/KE1	[−4.41; 4.79]	[−30.59; −14.65]	[15.03; 30.97]
MT2	[−5.98; 7.06]	[−43.06; −20.47]	[21.55; 44.14]
KI2/KE2	[−14.59; −0.70]	[−54.08; −30.02]	[14.74; 38.80]
Leg-spreading angles, °			
MT1	[1.65; 7.87]	[−16.03; −5.26]	[14.78; 25.54]
KI1/KE1	[1.12; 8.69]	[−20.41; −7.30]	[17.10; 30.21]
MT2	[−5.09; 5.86]	[−36.23; −17.26]	[18.04; 37.01]
KI2/KE2	[−9.01; 4.07]	[−46.21; −23.55]	[18.60; 41.26]
Distance- and height-based characteristics, percentage points			
DKS1	[−2.55; 3.21]	[−18.91; −8.95]	[9.60; 19.57]
DKS2	[0.28; 7.95]	[−21.54; −8.25]	[16.48; 29.77]
KHK1	[−8.37; 3.01]	[−40.72; −21.01]	[15.66; 35.37]
KI1 knee height	[−10.21; 3.27]	[−48.56; −25.20]	[18.26; 41.62]
KHK2	[−14.12; 1.82]	[−59.46; −31.84]	[19.54; 47.16]

## Appendix B. Normality Tests for Bland–Altman Differences

**Table A6:** Shapiro–Wilk normality tests for the Bland–Altman differences. Differences were calculated as video-based value minus 3D reference value.

Model	Characteristic	*n*	*W*	*p*	Normality
ViTPose-L	CH1	26	0.779	<0.001	Rejected
ViTPose-L	KI1	26	0.898	0.014	Rejected
ViTPose-L	CH2	26	0.874	0.004	Rejected
ViTPose-L	KI2	26	0.930	0.076	Not rejected
ViTPose-L	Femur angle MT1	27	0.916	0.031	Rejected
ViTPose-L	Femur angle KI1/KE1	27	0.978	0.806	Not rejected
ViTPose-L	Femur angle MT2	27	0.943	0.143	Not rejected
ViTPose-L	Femur angle KI2/KE2	27	0.916	0.032	Rejected
ViTPose-L	Leg-spreading angle MT1	27	0.943	0.143	Not rejected
ViTPose-L	Leg-spreading angle KI1/KE1	27	0.920	0.041	Rejected
ViTPose-L	Leg-spreading angle MT2	27	0.985	0.951	Not rejected
ViTPose-L	Leg-spreading angle KI2/KE2	27	0.931	0.073	Not rejected
ViTPose-L	DKS1	27	0.957	0.323	Not rejected
ViTPose-L	DKS2	27	0.974	0.713	Not rejected
ViTPose-L	KHK1	27	0.878	0.004	Rejected
ViTPose-L	KI1 knee height	27	0.960	0.362	Not rejected
ViTPose-L	KHK2	27	0.873	0.003	Rejected
RTMPose-L	CH1	26	0.826	<0.001	Rejected
RTMPose-L	KI1	26	0.962	0.432	Not rejected
RTMPose-L	CH2	26	0.917	0.038	Rejected
RTMPose-L	KI2	26	0.971	0.645	Not rejected
RTMPose-L	Femur angle MT1	27	0.964	0.443	Not rejected
RTMPose-L	Femur angle KI1/KE1	27	0.949	0.203	Not rejected
RTMPose-L	Femur angle MT2	27	0.834	<0.001	Rejected
RTMPose-L	Femur angle KI2/KE2	27	0.768	<0.001	Rejected
RTMPose-L	Leg-spreading angle MT1	27	0.903	0.016	Rejected
RTMPose-L	Leg-spreading angle KI1/KE1	27	0.989	0.989	Not rejected
RTMPose-L	Leg-spreading angle MT2	27	0.882	0.005	Rejected
RTMPose-L	Leg-spreading angle KI2/KE2	27	0.826	<0.001	Rejected
RTMPose-L	DKS1	27	0.829	<0.001	Rejected
RTMPose-L	DKS2	27	0.987	0.979	Not rejected
RTMPose-L	KHK1	27	0.838	<0.001	Rejected
RTMPose-L	KI1 knee height	27	0.918	0.035	Rejected
RTMPose-L	KHK2	27	0.803	<0.001	Rejected

## Appendix C. Results With and Without Anomaly Detection

**Table A7:** Paired comparison of anomaly detection disabled (AD off) and enabled (AD on), by pose-estimation model and movement-characteristic group. AD-off and AD-on values represent median subject-level absolute errors across all valid paired observations. (n) is the total number of valid paired observations, while $\left(n_{\neq 0}\right)$ is the number of nonzero paired differences included in the Wilcoxon signed-rank test. Differences were calculated as AD-off error minus AD-on error, so positive differences indicate lower error with anomaly detection enabled. (V) is the sum of ranks associated with positive differences. Raw exact (*p*)-values were adjusted using the Holm correction across all 16 comparisons.

Model	Movement-Characteristic Group	*n*	Changed Subjects	AD Off	AD On	Raw *p*	Holm *p*
MoveNet	Phase durations	26	9	0.056	0.048	0.734	1.000
MoveNet	Femur angles	25	7	8.779	9.371	0.688	1.000
MoveNet	Leg-spreading angles	23	9	8.961	8.695	0.570	1.000
MoveNet	Distance- and height-based characteristics	27	7	7.830	7.540	0.078	1.000
RTMPose-L	Phase durations	26	1	0.044	0.043	1.000	1.000
RTMPose-L	Femur angles	27	2	7.514	6.410	0.500	1.000
RTMPose-L	Leg-spreading angles	27	2	6.729	6.729	0.500	1.000
RTMPose-L	Distance- and height-based characteristics	27	1	6.086	5.690	1.000	1.000
ViTPose-L	Phase durations	26	1	0.043	0.043	1.000	1.000
ViTPose-L	Femur angles	27	2	6.720	6.720	1.000	1.000
ViTPose-L	Leg-spreading angles	27	3	6.216	6.216	0.250	1.000
ViTPose-L	Distance- and height-based characteristics	27	1	6.805	6.805	1.000	1.000
YOLO11x-Pose	Phase durations	26	6	0.072	0.067	0.156	1.000
YOLO11x-Pose	Femur angles	27	7	7.411	7.411	0.375	1.000
YOLO11x-Pose	Leg-spreading angles	27	10	7.856	9.919	0.557	1.000
YOLO11x-Pose	Distance- and height-based characteristics	27	5	7.308	7.077	1.000	1.000

## Appendix D. Pairwise Post-Hoc Comparisons for Significant Friedman Tests

**Table A8:** Pairwise Wilcoxon signed-rank post-hoc comparisons for the movement-characteristic groups with significant Friedman tests. The first-listed configuration in each comparison is the configuration with the lower median error. Positive matched-pairs rank-biserial correlations, therefore, indicate lower paired errors for the first-listed configuration. Holm-adjusted *p*-values were corrected across the six pairwise comparisons within each movement-characteristic group.

Movement-Characteristic Group	Comparison	*n*	*W*	Raw *p*	$p_{\mathrm{Holm}}$	$r_{\mathrm{rb}}$
Phase durations	ViTPose-L vs. YOLO11x-Pose	26	54	0.006	0.037	0.640
Phase durations	RTMPose-L vs. YOLO11x-Pose	26	72	0.015	0.074	0.557
Phase durations	ViTPose-L vs. MoveNet	26	71	0.072	0.286	0.439
Phase durations	MoveNet vs. YOLO11x-Pose	26	94	0.110	0.329	0.373
Phase durations	RTMPose-L vs. MoveNet	26	118	0.361	0.721	0.213
Phase durations	ViTPose-L vs. RTMPose-L	26	75	0.943	0.943	0.020
Femur angles	ViTPose-L vs. MoveNet	25	56	0.003	0.019	0.655
Femur angles	RTMPose-L vs. MoveNet	25	91	0.055	0.275	0.440
Femur angles	YOLO11x-Pose vs. MoveNet	25	96	0.075	0.302	0.409
Femur angles	ViTPose-L vs. RTMPose-L	25	144	0.634	1.000	0.114
Femur angles	ViTPose-L vs. YOLO11x-Pose	25	149	0.731	1.000	0.083
Femur angles	YOLO11x-Pose vs. RTMPose-L	25	158	0.916	1.000	−0.028

## Appendix E. Input-Framing Sensitivity Analysis

**Table A9:** Input-framing sensitivity analysis comparing the original full-frame configurations with detector-crop variants. Medians were calculated on the complete paired observations available for each comparison and may, therefore, differ from the four-configuration complete-case medians in Table 3. Positive $r_{\mathrm{rb}}$ values favor the configuration with the lower median error.

Model	Group	*n*	Full-Frame Median	Detector-Crop Median	*W*	Raw *p*	$p_{\mathrm{Holm}}$	$r_{\mathrm{rb}}$
MoveNet	Phase durations	26	0.056	0.064	157	0.882	1.000	0.034
MoveNet	Femur angles	24	9.125	9.623	143	0.855	1.000	0.047
MoveNet	Leg-spreading angles	23	8.961	9.917	96	0.211	1.000	0.304
MoveNet	Distance/height	27	7.830	6.811	160	0.500	1.000	0.153
YOLO11x-Pose	Phase durations	26	0.072	0.067	146	0.909	1.000	−0.027
YOLO11x-Pose	Femur angles	27	7.411	13.430	93	0.020	0.140	0.508
YOLO11x-Pose	Leg-spreading angles	27	7.856	13.966	64	0.002	0.015	0.661
YOLO11x-Pose	Distance/height	27	7.308	9.460	146	0.313	1.000	0.228
